# Viscosity and density measurements on the cytosol of human red blood cells

**DOI:** 10.1016/j.bpj.2025.07.002

**Published:** 2025-07-15

**Authors:** Thomas John, Karin Kretsch, Felix Milan Maurer, Steffen Michael Recktenwald, Lars Kaestner, Christian Wagner

**Affiliations:** 1 Experimental Physics, Saarland University, Saarbrücken, Germany; 2 Theoretical Medicine und Biosciences, Saarland University, Homburg, Germany; 3 Department of Physics and Materials Science, University of Luxembourg, Luxembourg, Luxembourg

## Abstract

We introduce an approach for determining the viscosity of the intracellular liquid, called cytosol, of human red blood cells (RBCs). This methodology combines measurements of the mass density distribution of RBCs and the viscosity of the cytosol relative to its water content. The density distribution is obtained through buoyant density centrifugation paired with cell counting. By correlating the Gaussian distribution of cell population densities with the viscosity-density relationship of the cytosol, we derive a log-normal distribution of the cytosol viscosity in healthy RBCs. The viscosity contrast $\lambda =\eta /\eta_{\text{plasma}}$, which is the ratio between viscosities of the RBC cytosol and blood plasma under physiological conditions, is found to have a mean value of $\bar{\lambda }=10$. This value is notably higher than those cited in existing literature for numerical simulations. The broad range of viscosity values stems from the gradual loss of water from RBCs over their 120-day lifespan. Our findings indicate that older RBCs exhibit more than twice the cytosol viscosity of younger cells, a critical factor for future theoretical studies of physiological conditions.

## Significance

The numerical prediction of cardiovascular diseases is a promising prospect in reducing the leading global risk factor for death. Simulating blood flow at the cellular level requires precise knowledge of the mechanical properties of red blood cells. The cytosol viscosity, as one critical parameter in cell dynamics, is still a subject of discussion. The cytosol, an almost saturated hemoglobin solution, undergoes changes in its water content over the 120-day lifespan of red blood cells, altering its viscosity. This study determines the distribution of cytosol viscosity in healthy human red blood cells experimentally by measuring their density and viscosity through a combined approach. These findings are essential for developing accurate numerical models of blood flow, both in health and disease.

## Introduction

Cardiovascular diseases are the main cause of death worldwide, and a significant amount of numerical work is dedicated to the prediction of related risk factors. Despite the complex non-Newtonian properties of blood (1,2,3), many medically driven numerical studies on patient-specific cardiovascular risk prediction simplify blood to a Newtonian fluid, like water (4,5). A comprehensive description of in vitro blood flow that is based on mechanical properties of the cellular components has been proven to be feasible, at least in systems of limited size (6,7,8,9). Significant progress has been made in predicting some stationary red blood cell (RBC) shapes in capillary flow (10,11), but the mechanical properties of blood cells are not fully understood yet. Given that RBCs constitute approximately 45% of blood volume (hematocrit), their properties are crucial for modeling blood flow accurately. RBCs are highly deformable objects with a constant surface/volume ratio, encapsulated by a membrane characterized by a specific bending rigidity and shear stiffness. Although the elastic constants of the membrane are documented (12,13,14), less is known about the viscosity η of the interior of the cell (cytosol) (15,16,17,18). The cytosol, free of a nucleus and organelles, consists mainly of hemoglobin, water, and numerous other proteins that are few in number compared to hemoglobin. In simulations, the viscosity of the cytosol is often scaled relative to the surrounding plasma. Early studies typically assumed $\eta /\eta_{\text{plasma}}=\lambda =1$ for simplicity (19), with later adjustments to more realistic values such as λ=5 (20), but rarely higher (21), though direct experimental data is still insufficient. Evidence from comparisons of experimental RBC shape oscillations in flow with numerical simulations suggests that even higher values, λ⩾10, may predict RBC flow more accurately (22).

The average lifespan of RBCs in the human body is 120 days (23,24,25), and during this period, the membrane properties, such as the shear modulus, change (26). A progressive release of vesicles with a higher content in lipids than proteins reduces the membrane surface area, and the cell loses a small amount of water (27). This results in a significant increase in the cytosol viscosity. Our hypothesis is that a healthy population of RBCs, containing cells of various ages, exhibits a broad distribution of cytosol viscosities. We demonstrate that our method can determine not only the average cytosol viscosity of RBCs but also the distribution of viscosities within the population. To obtain the cytosol viscosity contrast distribution, pdf(λ), we first introduce a technique of measuring the mass density distribution, ${\text{pdf}}_{\rho }\left(\rho \right)$, of the RBCs and then combine this with the experimentally determined viscosity-density relation, η(ρ). The RBC mass density distribution is obtained by isopycnic centrifugation, utilizing self-forming Percoll (Cytiva Sweden, Uppsala, Sweden) gradients (28). The volumetric RBC concentration and mass density of samples extracted from different positions in the gradient are measured systematically. Given the thinness of the membrane (50 nm), the total cell density is equivalent to the density of the cytosol (29,30). Additionally, we measure the viscosity of the cytosol extracted from RBCs with a rolling-ball rheometer in a different experiment. RBC suspensions are ultracentrifuged, and the cell membranes in the resulting pellet are destroyed using ultrasound. Membrane residues are then separated by further centrifugation. In this process, it is not possible to control the amount of remaining external water from the washing steps. Therefore, we again measure the viscosity and simultaneously the mass density for a dilution series to obtain the viscosity of the cytosol as a function of its density, η(ρ). To verify how variations in physiological osmolarity might affect the water concentration and thus the cytosol viscosity, we also prepare hypertonic samples of higher osmolarities. Our measurements show that the mean viscosity of the cytosol of healthy RBCs is $\bar{\lambda }=10$ and that the viscosity of old cells is more than twice as high compared to young cells. Mathematically, our data are well described by a rather broad log-normal distribution ranging from λ=5 to λ=20 and a median of 10.

## Materials and methods

### Sample preparation for RBC density distribution measurements

**Figure 1:**
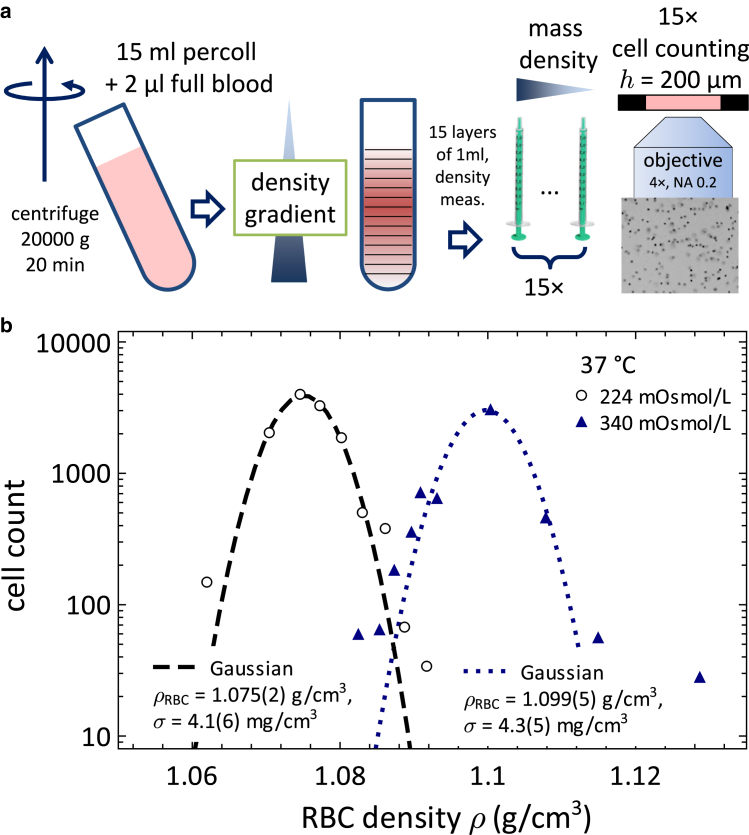
(*a*) Method for determining the cell density distribution. A small volume of whole blood is added to a Percoll medium, which is a colloidal silica suspension that forms a density gradient under gravitational force. After centrifugation, the cells are positioned along the tube according to their density. Fifteen aliquots of 1 mL are extracted, and both the suspension density and the cell count per volume are determined; the cropped image shows an area of 600 × 500 *μ*m^2^. All density measurements are conducted at 37°C, as detailed in the materials and methods section. (*b*) Illustrations of Gaussian fits for RBC mass density distributions at two different osmolarity extremes. The average RBC mass density $\rho_{\text{RBC}}$ increases with the suspension osmolarity, whereas the distribution width σ is unchanged. See Fig. S1.

Density gradient media at various osmolarities and densities are prepared by diluting a Percoll stock solution (Cytiva Sweden, $\rho_{0}=$ 1.130 g/cm^3^) with phosphate-buffered saline (PBS) at various concentrations. The osmolality is measured with a freezing-point osmometer (Knauer Osmometer Automatic, accuracy ±10mOsmol/kg). In the paper, we convert the measured osmolalities to osmolarities assuming a liquid density of 1 kg/L. The pH is adjusted to 7.4 using HCl/NaOH. A small sample of approximately 2 *μ*L of full blood extracted by finger puncture is homogeneously suspended in tubes (Beckman-Coulter, Carlsbad, California, 16  × 104 mm) containing 15 mL of density gradient media. This strong dilution prevents the aggregation of cells (31). The contribution of plasma and other types of cells in the suspension is negligible. The suspension is then centrifuged at 20,000 × *g* for 20 min. After centrifugation, 15 samples of 1 mL each are carefully extracted using a motorized syringe pump, layer by layer (31). For each sample, the density is measured with a density meter (Anton Paar DMA 5000M, Anton Paar, Ashland, Virginia). The number of cells per volume is determined using a Malassez Counting Chamber of 200 *μ*m height. Before filling the chamber, the individual syringes/layers are shaken to achieve a homogeneous distribution in the syringe. The cells are isopycnic in the Percoll and do not sediment; thus, an image stack for cell counting is captured over the entire chamber height and analyzed using a homemade MATLAB script. Blue illumination is used for high-contrast absorption imaging with a Nikon microscope. The objective was a 4× NA 0.2, and the camera was Imaging Source DMK33UP5000. The field of view was 3.1×2.5 mm and 25 times larger, as illustrated in the cropped example in Fig. 1 *a*.

### Sample preparation for viscosity measurements

Fresh blood is drawn from healthy donors into tubes containing EDTA as an anticoagulant. Samples are centrifuged for 5 min at 6000 × *g* in a fixed angle rotor (Hermle Germany Z36HK, rotor 221.22, Hermle Germany, Gosheim, Germany). The supernatant plasma and “buffy coat” are removed. The remaining RBC pellet is then diluted with PBS (Gibco/Thermo Fisher Scientific, Waltham, Massachusetts; pH 7.4, ${\text{Ca}}^{++}$ and ${\text{Mg}}^{++}$ free) and centrifuged again for 5 min at 6000 × *g*. This washing procedure is repeated three times. In certain samples, a PBS solution with elevated osmolarity (400 mOsmol/L) is used to extract additional water from the cells. Finally, the samples are centrifuged at 40,000 × *g* for 10 min to obtain a maximally packed RBC pellet. The supernatant PBS is removed, and the remaining pellet is subjected to ultrasound treatment to destroy the cell membranes. A rod-shaped device with a diameter of 2 mm and a power density of 600 W/cm^2^ (Hielscher, Tetlow, Germany, UP100H) is employed. During the two 5-min ultrasound treatments, the RBC pellet is cooled in a water bath, and an interval mode is used to avoid overheating. A subsequent examination using capillary electrophoresis shows no changes in hemoglobin after ultrasound treatment. The sample is then centrifuged again at 40,000 × *g* for 60 min. A small sediment content of less than 2 vol % is observed, which we identify as membrane residues. No membrane residues are observed under microscopic examination in the cytosol solution drawn from the middle section of the tubes, which is used in subsequent experiments. We opted not to use substances such as toluene to dissolve the membranes, to minimize the impact of aggressive chemicals on the cytosol.

### Ethical statement

Blood collection is performed following the declaration of Helsinki and is approved by the ethics committee of “Ärztekammer des Saarlandes,” permit number 51/18.

## Results

### RBC density distribution

We determine the mass density distribution of RBCs under various osmotic conditions through isopycnic centrifugation, using self-forming Percoll gradients (see Fig. 1 *a*). To prevent cell aggregation and band formation, only a small amount of blood/RBCs is used (31), which also allows us to count the number of cells per density layer microscopically. Fig. 1 *b* displays the RBC density distributions at two distinct (unphysiologically low and high) osmolarities. The cell mass density distribution shifts toward lower or higher mean densities due to water loss or uptake while maintaining a constant solid content within the cytosol for individual cells. The density distribution width σ= 5(2) mg/cm^3^, arising from the variance in densities between young and old cells, remains constant across all osmolarities (see Fig. S1). The notation of uncertainties in brackets follows the guide to the expression of uncertainty in measurement (GUM) (32).

**Figure 2:**
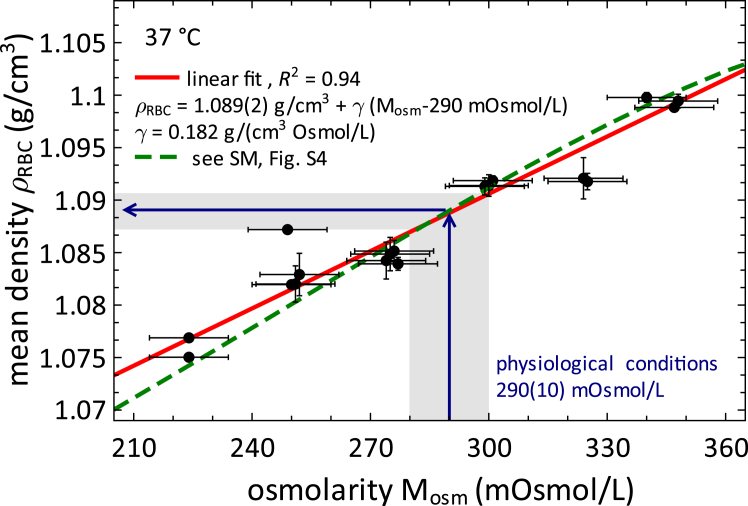
Mean densities of RBCs at different osmolarities. The mean density increases almost linearly with the osmolarity, as indicated by the solid red line. The error bars for the osmolarity are device uncertainties. The error bars in the mean density are statistical errors derived from fitting the cell counting data set to a Gaussian distribution; some are in order of symbol size. The gray area highlights the range of physiological osmolarities, with arrows indicating their mean. The red line is a linear fit to our experimental data, whereas the dashed green line is derived from experimental data on cell volumes reported in (33,34). See the discussion section and supporting material.

A linear relationship is fitted between extracted mean density and preparation osmolarity (see Fig. 2). At physiological conditions of 290 mOsmol/L and 37°C, the mean density is $\rho_{\text{RBC}}=$ 1.089(2) g/cm^3^. The dashed green line reflects a parameter-free calculation, based on the mean value at 290 mOsmol/L and the mean cellular volume (MCV) as a function of the osmolarity (see the discussion section and Fig. S4). The temperature dependency for below 37°C is also measured (see Fig. S3). In addition, typically, the normal daily variation in plasma osmolarity for a healthy individual is less than 2 mOsmol/L (35).

### RBC cytosol viscosity distribution

**Figure 3:**
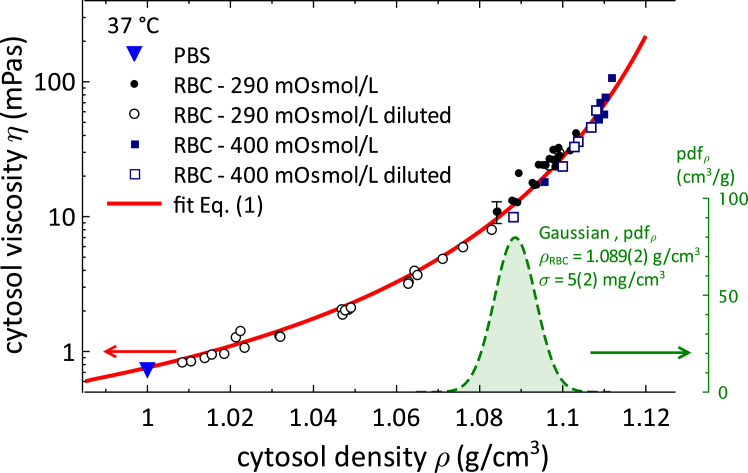
Cytosol viscosity at 37°C for undiluted (*solid symbols*) and diluted (*open symbols*) samples prepared with 290  and 400 mOsmol/L, respectively. The solid line represents a fit of function (Eq. 1). The Gaussian distribution of RBC densities ${\text{pdf}}_{\rho }$ under physiological conditions is depicted to highlight the relevant range (dashed green line). The error bar shows the device uncertainties.

We now turn to the viscosity measurements of the RBC cytosol at various degrees of dilution, accompanied by precise density measurements. To extract the cytosol from RBCs, we washed the cells three times with PBS buffer and then centrifuged at 40,000 × *g* after the final washing, yielding a compact cell pellet. This pellet is treated with ultrasound to break up the cell membranes, and membrane residues are removed subsequently, as detailed in the materials and methods section. Despite the dense packing of the pellet, some PBS remains in the sample, leading to variability in the viscosity measurements (17). This residual PBS, which cannot be controlled in our preparation method, significantly impacts the viscosity of the sample. The viscosity of the cytosol in RBC changes dramatically with its density, minor hemoglobin concentration variations respectively. The hemoglobin concentration in RBCs is close before its maximum solubility, and the viscosity increases super-exponentially as illustrated in Fig. 3. However, the solid content concentration can be accurately determined through density measurements. We also diluted several samples with PBS to establish a viscosity-density relation across the range from pure PBS to undiluted cytosol. Additionally, washing cells in hyperosmotic solutions, such as 400 mOsmol/L-PBS, results in water loss from the cells before ultrasound treatment, leading to slightly higher densities and significantly higher viscosities. Data from three healthy donors (two males and one female) showed no significant variation.

The cytosol is predominantly a mixture of hemoglobin, proteins, and water. Based on the approximation that hemoglobin molecules are spherical, Ross and Minton introduced a semiempirical, hard quasi-spherical model (36,37) for the viscosity of hemoglobin solutions. Adapting this model to density units, we use a similar relation to fit the viscosity:

(1) $$\eta \left(\rho \right)=\eta_{\text{PBS}}\;\exp\left[\frac{\alpha \left(\rho -\rho_{\text{PBS}}\right)}{1-\alpha \beta \left(\rho -\rho_{\text{PBS}}\right)}\right]\text{.}$$

This model equation contains only two parameters, with best-fit values α= 15.6(4) cm^3^/g and β=0.34(2). Eq. 1 predicts a divergence in viscosity at a density of 1.183 g/cm^3^, corresponding to a solid content concentration of 69 wt %. However, we only achieved solutions with concentrations up to 45 wt %.

The mass density of RBCs is determined by their cytosol composition, allowing us to combine the Gaussian distribution of cell density ${\text{pdf}}_{\rho }\left(\rho \right)$ with the viscosity-density relation η(ρ). We translate this to the viscosity contrast, $\lambda =\eta /\eta_{\text{plasma}}$, the relevant reduced quantity in theoretical hemodynamic analysis. The average plasma viscosity is $\eta_{\text{plasma}}\left(37{}^{\circ}C\right)=1.25\left(15\right)\text{mPas}$ (see Fig. S2). The probability transformation to the viscosity contrast distribution pdf(λ) is derived analytically using the inverse function of Eq. 1 and the parameters $\rho_{\text{RBC}}$ and σ of the Gaussian distribution of RBC mass densities:

(2) $$\rho \left(\eta \right)=\rho_{\text{PBS}}+\frac{\ln\left(\eta /\eta_{\text{PBS}}\right)}{\alpha +\alpha \beta \;\ln\left(\eta /\eta_{\text{PBS}}\right)}\text{,}$$

$$\frac{d\eta \left(\rho \right)}{d\rho }=\frac{\alpha \;\eta_{\text{PBS}}}{{\left(1-\alpha \;\beta \;\left(\rho -\rho_{\text{PBS}}\right)\right)}^{2}}\times$$

(3) $$\times \exp\left[\frac{\alpha \left(\rho -\rho_{\text{PBS}}\right)}{1-\alpha \;\beta \;\left(\rho -\rho_{\text{PBS}}\right)}\right]\text{,}$$

(4) $${\text{pdf}}_{\rho }\left(\rho \right)=\frac{1}{\sqrt{2\pi \sigma^{2}}}\exp\left[\frac{-{\left(\rho -\rho_{\text{RBC}}\right)}^{2}}{2\sigma^{2}}\right]\text{,}$$

(5) $${\text{pdf}}_{\eta }\left(\eta \right)={\text{pdf}}_{\rho }\left(\rho \left(\eta \right)\right){\left|\frac{d\eta \left(\rho \right)}{d\rho }\left(\rho \left(\eta \right)\right)\right|}^{-1},\text{and}$$

(6) $$\text{pdf}\left(\lambda \right)=\eta_{\text{plasma}}{\text{pdf}}_{\eta }\left(\eta_{\text{plasma}}\lambda \right)\text{.}$$

**Figure 4:**
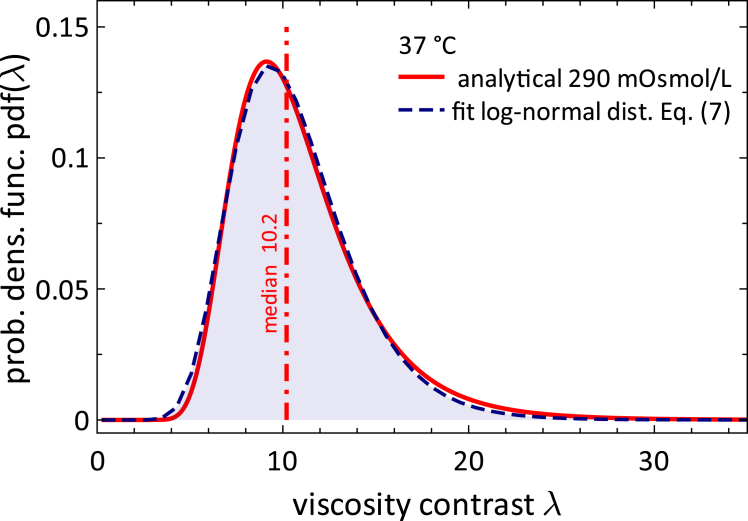
The probability density function of the cytosol viscosity contrast at physiological conditions (*solid red line*). It can be approximated by a log-normal distribution (Eq. 7; *dashed blue line*). The median is given by $\mu^{*}=\eta \left({\bar{\rho }}_{\text{RBC}}\right)/\eta_{\text{plasma}}=10.2$, and the fitted geometric standard deviation is $\sigma^{*}=1.35$.

This yields the final result, the probability density function of the cytosol viscosity contrast pdf(λ), shown in Fig. 4. Near the mean cell density $\rho_{\text{RBC}}$, the viscosity of the cytosol follows an exponential function, transforming the Gaussian distribution of RBC mass density into a log-normal distribution (38), as depicted in Fig. 4:

(7) $$\text{pdf}\left(\lambda \right)\;\approx \;\frac{1}{\lambda \;\ln\left(\sigma^{*}\right)\sqrt{2\pi }}\exp\left[-\frac{\ln^{2}\left(\lambda /\mu^{*}\right)}{2\;\ln^{2}\left(\sigma^{*}\right)}\right]\text{.}$$

At 22°C, the plasma viscosity increases by a factor of 1.4 compared to 37°C (see Fig. S2). The cytosol viscosity at mean cell density increases by a factor of 1.55. Consequently, the viscosity contrast λ increases by 10% at room temperature. Notably, the composition of blood plasma can vary significantly between individuals and times of day, with plasma viscosity ranging from 1.1  to 1.5 mPa s (39,40,41), thus impacting λ considerably.

**Figure 5:**
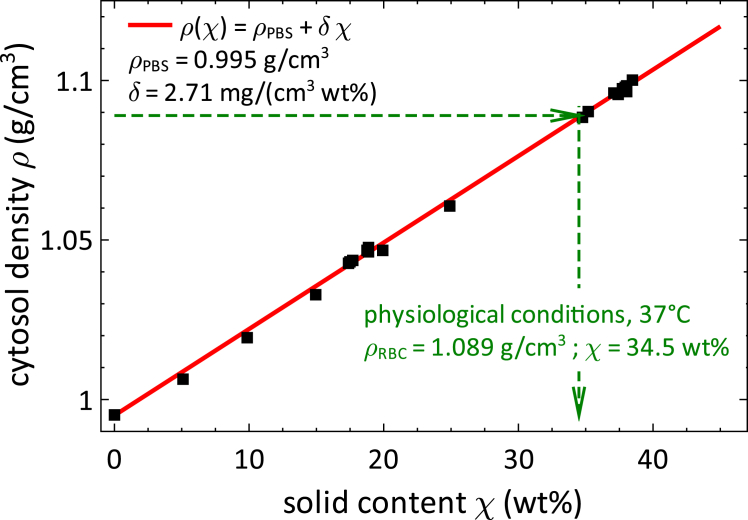
The dependency of densities ρ on solid content χ in wt % in the cytosol at 37°C determined by dry-freezing shows a linear relation. The mean RBC density corresponds to a solid content of 34.5 wt%.

**Figure 6:**
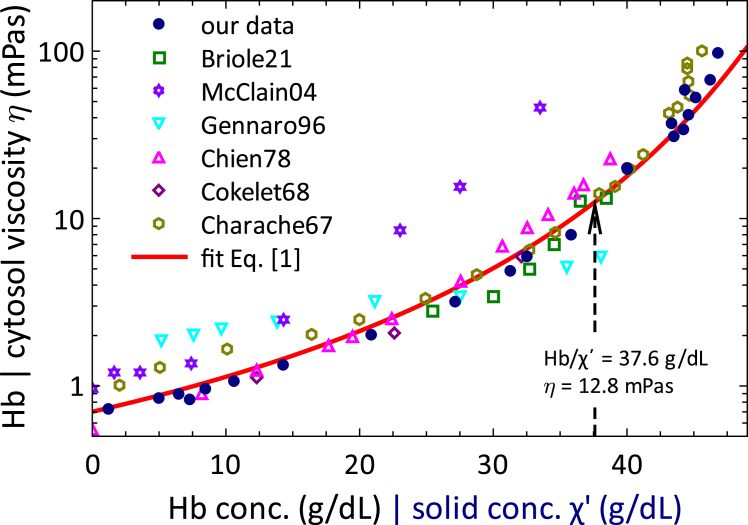
Viscosity data obtained in this study for various cytosol solid content concentrations $\chi^{\prime }$ are compared with existing literature data for different concentrations of human hemoglobin solutions (15,17,44,45,46,47). The fit is applied solely to our data (see Figs. 3 and 5).

Some of our data have been obtained in a similar way to the literature before, and for a direct comparison, we need to look very carefully at the relationship between hemoglobin concentration in the cytosol and mass density. To determine the solid content, we freeze dry the samples after measuring their weight and density. From this, we determine the wt % of the sample as a function of its density as a linear relation (see Fig. 5). An extrapolation to χ=100wt% yields a density of 1.27 g/cm^3^. This is in reasonable agreement with protein densities reported in the literature (42), with a molecular weight for the hemoglobin of 65 kDa. At our mean density $\rho_{\text{RBC}}$ of the cytosol at 37°C and 290 mOsmol/L, we obtain a concentration of χ=34.5wt%. This can be translated into $\chi^{\prime }=\rho_{\text{RBC}}\times \chi =37.6$ in unit g/dL. This value is slightly higher than the reference range for blood tests of 32–36 g/dL of the mean cellular hemoglobin concentration (43). Whereas the latter is determined by optical spectroscopy and sensitive to hemoglobin only, our method would also take the small amount of other cytosol proteins into account. With this, our viscosity measurements can be compared with the literature data, and we find a good agreement over the full range of possible concentrations (15,17,44,45,46) (Fig. 6). However, only the tight coupling with density measurements enables unambiguous connections to cytosol viscosity distributions for healthy RBC populations.

## Discussion

RBCs have an average residence time in the blood circulation of 120 days, with a range of 70–140 days (23). It is well known that as the cells age, they lose water and, consequently, become denser (24). This results in a density distribution ${\text{pdf}}_{\rho }\left(\rho \right)$ of cells in healthy individuals, though this distribution is disrupted in various blood diseases. For example, dense, dehydrated RBCs are a characteristic feature of sickle cell disease, and a comprehensive study on RBC densities, including both healthy and sick patients, can be found in (48). This study employed the phthalate density-distribution technique (49) and reported a mean density of 1.095 g/cm^3^, aligning with our values when accounting for a 5 mg/cm^3^ increase from 20°C to 37°C. The reported standard deviation in this study of 3 mg/cm^3^ is slightly lower than our value. Another method, presented in (50), used resonance techniques on a microfluidic chip to determine the MCV and mass distribution of RBCs. This approach reported a mean density of 1.1 g/cm^3^ and a standard deviation of 5 mg/cm^3^. The standard deviation matches ours, but the mean density, even when temperature corrected, remains significantly higher, particularly considering its impact on viscosity.

Our data for various osmolarities can be compared with the measurements of MCV (33,34), which has also been determined for different osmolarities. These changes result in either a gain or loss of water, allowing us to deduce a density relationship, as detailed in Fig. S4: $\rho \left(M_{\text{osm}}\right)=\rho_{H2O}+\Delta \rho \times V\left(290\;\text{mOsmol}/L\right)/V\left(M_{\text{osm}}\right)$, with the measured density difference of RBCs versus PBS at 290 mOsmol/L being Δρ= 0.094 g/cm^3^. This estimation aligns excellently with our measurements, as depicted by the dashed green line in Fig. 2.

Although the literature reports on viscosity measurements of extracted cytosol or hemoglobin-water mixtures (Fig. 6), a definitive statement about the mean and distribution of the viscosity of actual RBC cytosol was missing. Briol et al. (15,16) proposed an innovative approach to directly measure the latter using fluorescent viscometric probes within the RBCs. However, their method only allows for determining relative differences between individual RBCs. Our approach utilizes macroscopic samples of a few milliliters to determine viscosity, thereby avoiding the challenges associated with obtaining accurate data through microrheological methods, especially in confined samples (51,52). Nevertheless, a closer look at the solid content in the cytosol is necessary. Our value of 37 g/dL is approximately 7% higher than the tabulated mean cellular hemoglobin concentration of 32–36 g/dL (43), likely attributable to the presence of additional proteins in the cell. This discrepancy may also explain why recent numerical studies use a viscosity contrast of λ=5, corresponding to the viscosity contrast measured spectroscopically in the cells. A potential critique of our method could be the concern that ultrasonic treatment of the pellet might not only break up membranes but might also cause portions of them to dissolve, which might not settle during subsequent centrifugation. However, this issue likely does not significantly affect the determined relationship η(ρ), as any increase in viscosity due to additional proteins or lipids would be compensated by the corresponding increase in cytosol density (30). The ultimate viscosity distribution of the liquid within the cells is derived by combining η(ρ) with the measured RBC density distribution.

## Conclusion

Any predictive theoretical or numerical statement on a specific vascular flow situation relies heavily on the physical parameters of RBCs, the most abundant cells in blood. In addition to the elastic constants of the membrane, the cytosol viscosity, or viscosity contrast λ, stands out as a crucial parameter. For future simulations, we recommend using λ=10 or even considering a log-normal distribution for the viscosity contrast. Although there is an ongoing debate regarding whether the dissipative effects of cytosol and membrane viscosities can be distinguished, our precise measurement of the former should contribute to resolving this issue.

## Data availability

All data for this manuscript can be found at https://doi.org/10.1016/j.bpj.2025.07.002.

## Acknowledgments

We acknowledge funding by the 10.13039/501100001659German Research Foundation (DFG) WA1336/12-2.

## Author contributions

T.J., L.K., and C.W. designed research; T.J., K.K., F.M.M., and S.M.R. performed research; T.J. analyzed data; and T.J., K.K., F.M.M., S.M.R., L.K., and C.W. wrote the paper.

## Declaration of interests

The authors declare no competing interests.

## Supporting material

Document S1. Figures S1–S6 and supporting material

Data S1. Primary data and programms

Data S2. Primary data and programms description

Document S2. Article plus supporting material
