# Supplementary material for: Viscosity and density measurements on the cytosol of human red blood cells
Source: Biophys J. 2025 Jul 15;124(16):2668–76. doi: 10.1016/j.bpj.2025.07.002 (PMC12414699; doi:10.1016/j.bpj.2025.07.002)
Supplement: Supplementary file 1 [file mmc1.pdf]

Biophysical Journal, Volume *124*

Supplemental information

Viscosity and density measurements on the cytosol of human red blood cells

Thomas John, Karin Kretsch, Felix Milan Maurer, Steffen Michael Recktenwald, Lars Kaestner, and Christian Wagner

Supplementary Material: Viscosity and density measurements on the cytosol of human red blood cells

Thomas John[1,*], Karin Kretsch[1], Felix Milan Maurer[1], Steffen Michael Recktenwald[1], Lars Keastner[1,2], and Christian Wagner[1,3]

[1]Experimental Physics, Saarland University, D-66123 Saarbrücken, Germany
[2]Theoretical Medicine und Biosciences, Saarland University, D-66421 Homburg, Germany
[3]Department of Physics and Materials Science, University of Luxembourg, L-1511 Luxembourg, Luxembourg
[*]Correspondence: thomas.john@uni-saarland.de

A SUPPLEMENTARY MATERIAL

A.1 Standard deviation of the RBC density distribution

The fit of Gaussian distributions to describe the density distribution of RBCs yields the mean density ρ_{RBC} (Fig. 2 in the main article) and the standard deviation σ. The mean density changes with osmolarity, whereas the width of the distribution remains constant, see Fig. S1.

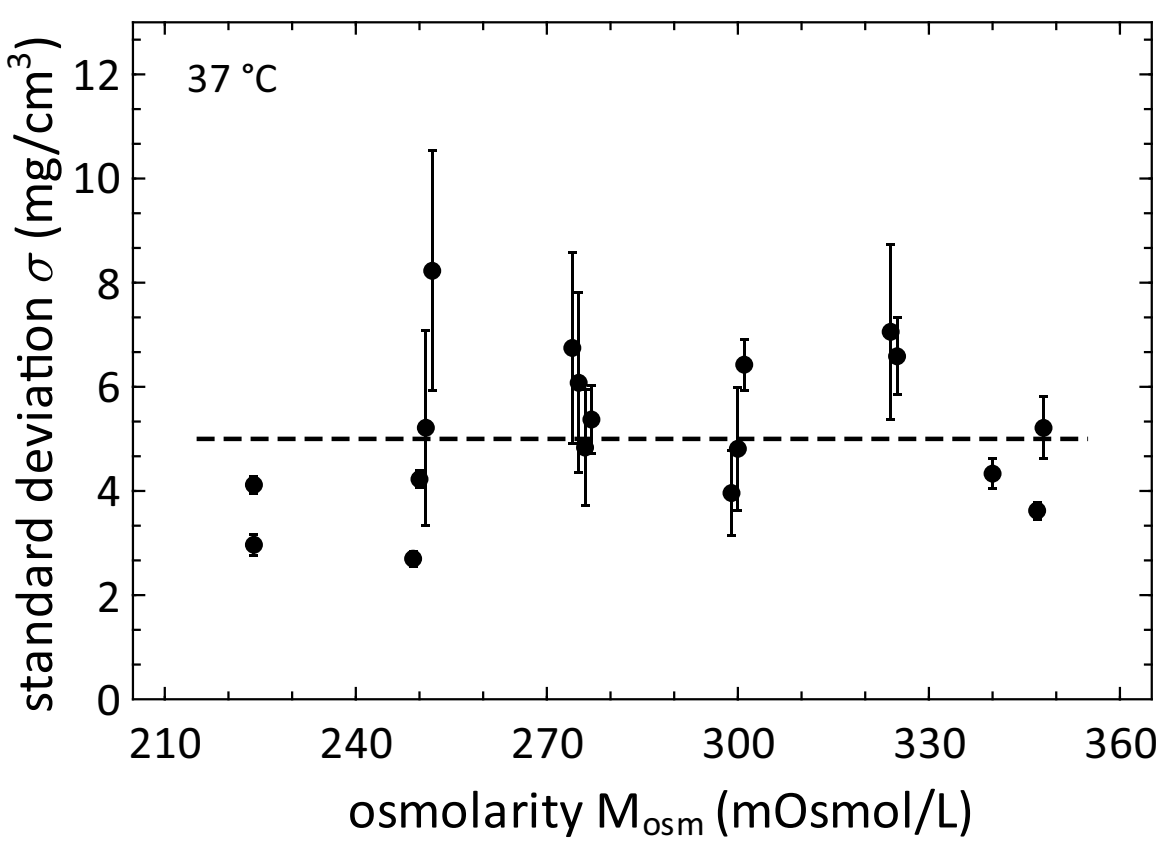

Figure S1: Standard deviation of RBC mass density distribution from cell counting in Percoll™ density gradients as a function of the osmolarity of the suspension at 37 °C. The standard deviation $\sigma = 5(2)\ \mathrm{mg/cm^3}$ of the Gaussian of the RBC mass density does not change with osmolarity, as represented by the dashed line.

A.2 Plasma and cytosol viscosity below body temperature

We present plasma and cytosol viscosity data from room to body temperature (Figures S2 and S3). We find that the viscosity contrast λ at 22 °C is 10% greater than at 37 °C, as the viscosity of the plasma increases by a factor of 1.4 and that of the cytosol by 1.55.

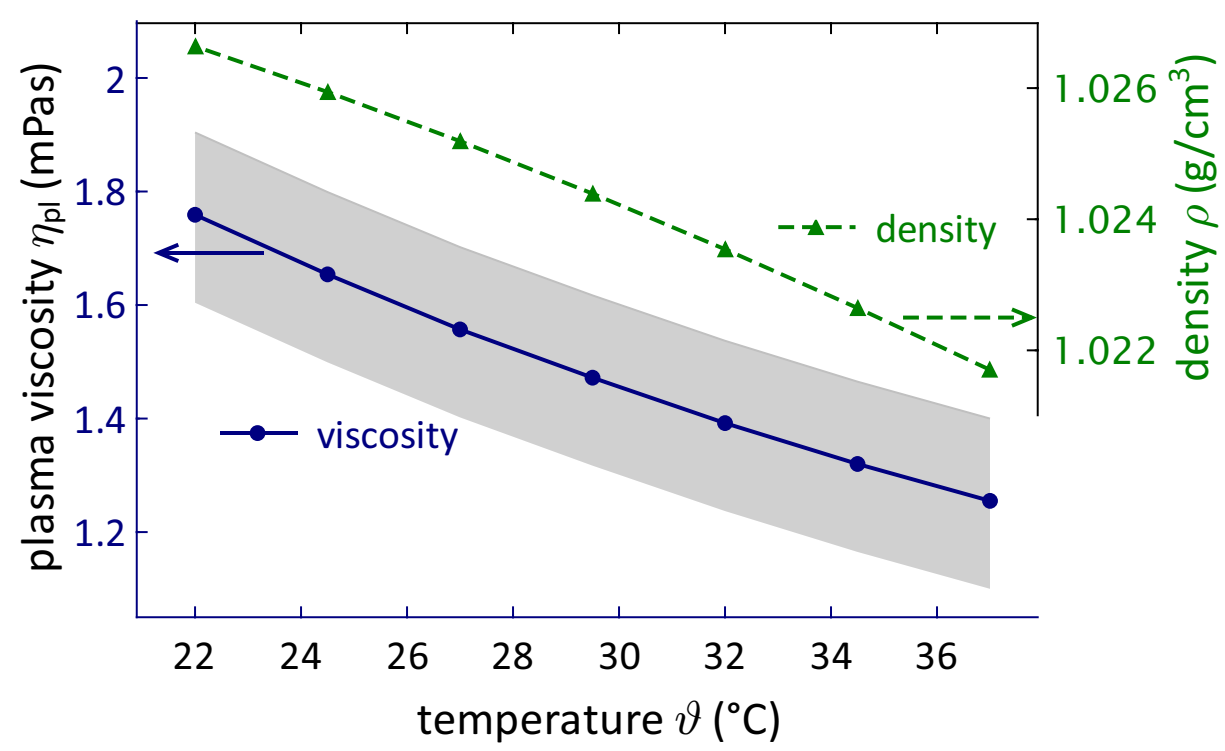

Figure S2: Viscosity and density of plasma as function of temperature. The viscosity can vary by 10% between different donors, shown as a gray area. We find a ratio between room and body temperature of $\eta_{22\,^{\circ}\mathrm{C,pl}}/\eta_{37\,^{\circ}\mathrm{C,pl}} = 1.4$.

A.3 Mean cellular volume (MCV) as function of osmolarity

The RBC membrane is permeable to water. A change in the surrounding liquid osmolarity leads to swelling or shrinking of the cells due to water intake/loss. A polynomial of second order is fitted to the mean cellular volume (MCV), reported in Refs. (1, 2) in order to estimated the density change with osmolarity (Fig. S4, cp. green dashed line in Fig. 2 in the main article).

A.4 The cytosol is a Newtonian fluid

We measure the viscosity of cytosol samples in steady shear using a stress-controlled rheometer (MCR702, Anton Paar,

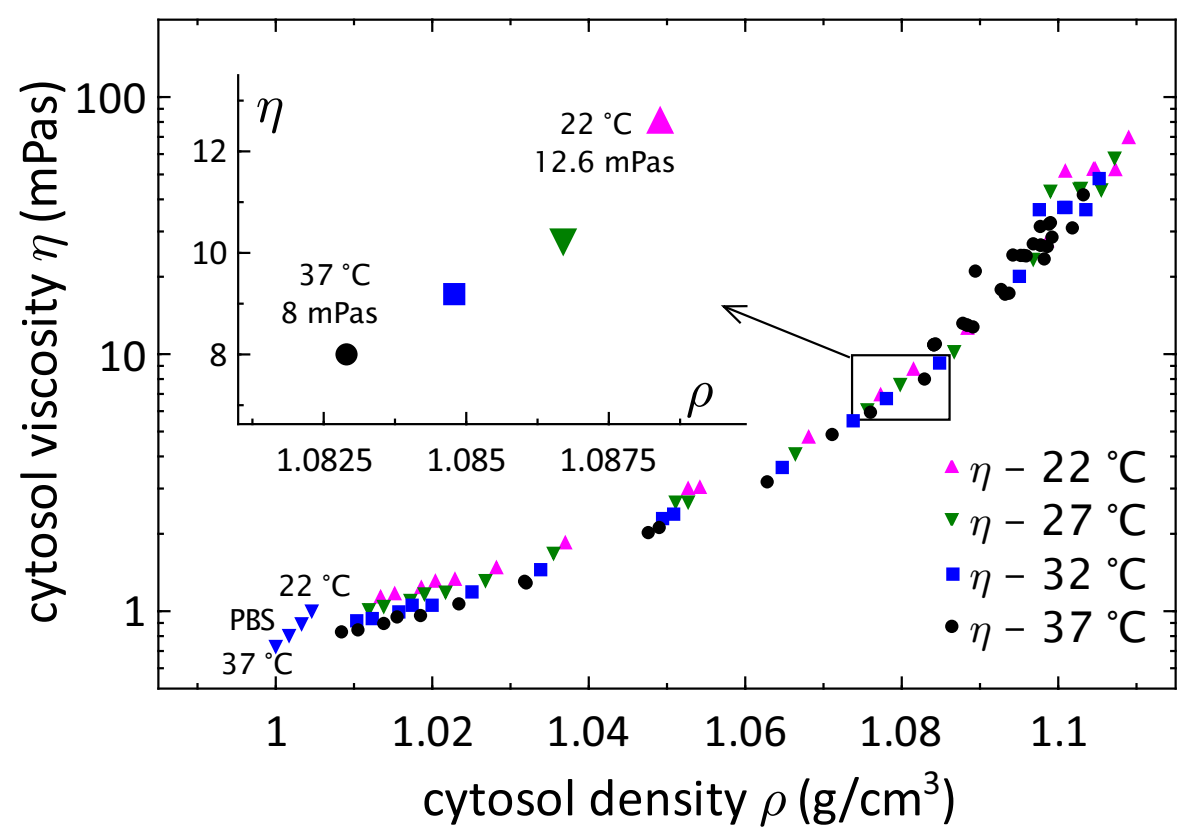

Figure S3: Cytosol viscosity at four different temperatures for varying densities. With decreasing temperature, density and viscosity increase. The density at 22 °C is 0.5% higher than at 37 °C, for PBS as well as for undiluted cytosol. The inset shows data for one sample at mean RBC density. For the cytosol we find a ratio between room and body temperature of $\eta_{22\,°\mathrm{C,cyt}}/\eta_{37\,°\mathrm{C,cyt}} = 1.55$.

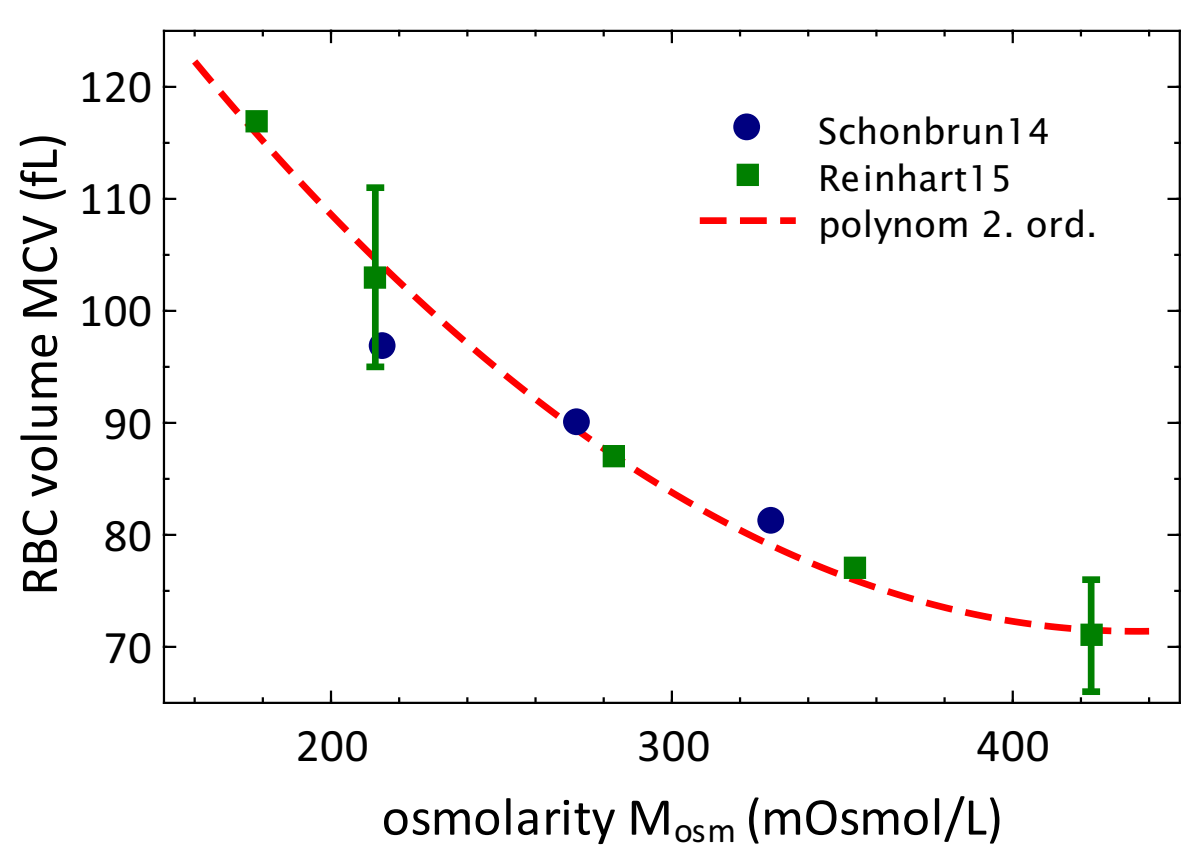

Figure S4: Mean cellular volume (MCV) of RBCs as function of the osmolarity, taken from Refs. (1, 2). We fit a second order polynomial to the data, it gives $V(\mathrm{M_{osm}}) = 198\,\mathrm{fL} - 0.58\,\mathrm{fL}/(\mathrm{mOsmol/L})\times\mathrm{M_{osm}} + 6.6\times10^{-4}\,\mathrm{fL}/(\mathrm{mOsmol/L})^2\times\mathrm{M_{osm}}^2$.

Graz, Austria) equipped with a coaxial cylinder geometry (CC20, Anton Paar, Graz, Austria, inner radius 10 mm, radius ratio of 1.1). Before the ultrasound treatment, one sample is prepared at 300 mOsmol/L and the second of hypertonic condition of 400 mOsmol/L to extract additional water from the RBC. Although the cytosol contains many proteins, it shows a Newtonian-like behavior in steady shear (Fig. S5). Especially the solution of physiological osmolarity exhibits a constant shear viscosity, but also the more viscous one does not show a pronounced shear-thinning, similar to the Newtonian reference fluid.

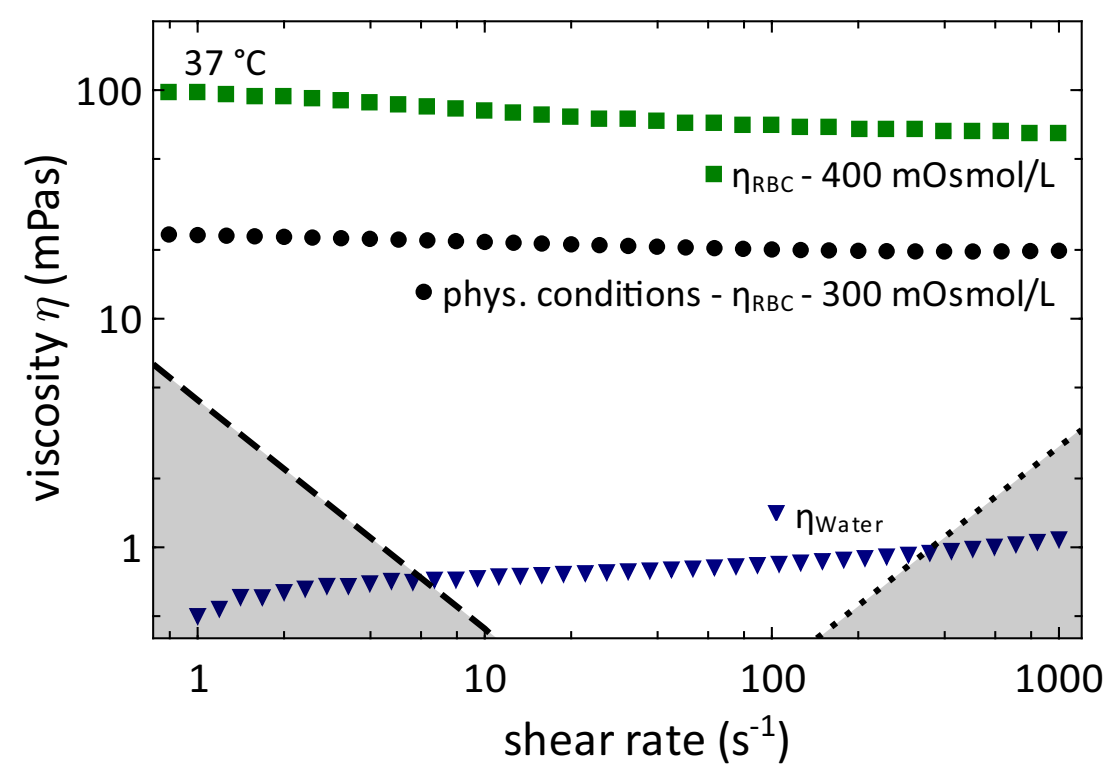

Figure S5: Steady shear rheology of two cytosol samples and the Newtonian reference fluid. Dashed and dotted black lines show the lower torque limit of the device and the onset of flow instabilities under steady shear measurements in the cylinder geometry, respectively (3).

A.5 Viscosity contrast at lower and higher osmolarities

The range of healthy physiological plasma osmolarities is between 280 and 300 mOsmol/L. These two extremes correspond to Gaussian distributions of the RBC density with a mean of 1.087 g/cm^3 and 1.091 g/cm^3 respectively, (cp. Fig. **??** in the main article). Those small density changes lead to a change of the cytosol viscosity, and even without considering differences between individual plasma viscosities, to a shift of the median of the log-normal distribution of the viscosity contrast. This is shown exemplarily in Fig. S6. The median of the log-normal distribution is given by the viscosity of the cytosol at the mean RBC density divided by the plasma viscosity $\mu^* = \eta(\bar{\rho}_{\mathrm{RBC}})/\eta_{\mathrm{plasma}}$.

A.6 Additional files

We also submitt a folder containing all figures as pdf and the raw data. The raw data are in .xls/.xlsx-Excel, .qti as QtiPlot files, a demo can be downloaded from the webside https://www.qtiplot.com/, .nb as Mathematica file, a viewer can be downloaded from https://www.wolfram.com/player/

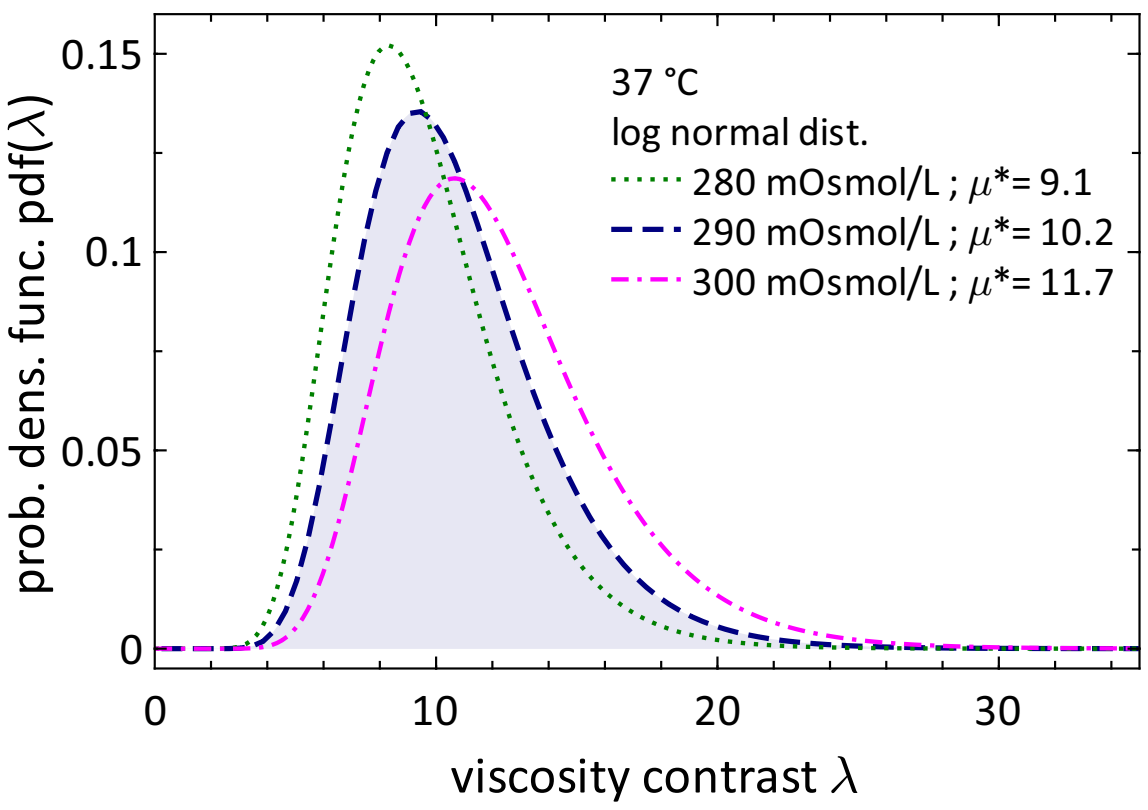

Figure S6: The log-normal distribution of the viscosity contrast is shifted by a smaller/larger osmolarity due to changes in the RBC density. The corresponding median is given in the legend, whereas the geometric standard deviation is constant at $\sigma* = 1.35$.

and a .m file as Matlab file. Those files includes comments about the data analysis. The raw images of the recorded image sequences to count the number of cells for a given density sample for Fig. 2 in the main article is not included, it is more than 2 Tbyte. The files can be provided on request to the first author.

REFERENCES

1. Schonbrun, E., R. Malka, G. Di Caprio, D. Schaak, and J. M. Higgins, 2014. Quantitative absorption cytometry for measuring red blood cell hemoglobin mass and volume: Quantitative Absorption Cytometry. *Cytometry Part A* 85:332–338. https://onlinelibrary.wiley.com/doi/10.1002/cyto.a.22450, number: 4.

2. Reinhart, W. H., N. Z. Piety, J. S. Goede, and S. S. Shevkoplyas, 2015. Effect of osmolality on erythrocyte rheology and perfusion of an artificial microvascular network. *Microvascular Research* 98:102–107. https://linkinghub.elsevier.com/retrieve/pii/S0026286215000114.

3. Ewoldt, R. H., M. T. Johnston, and L. M. Caretta, 2015. Experimental Challenges of Shear Rheology: How to Avoid Bad Data. *In* S. E. Spagnolie, editor, Complex Fluids in Biological Systems, Springer New York, New York, NY, 207–241. https://link.springer.com/10.1007/978-1-4939-2065-5_6, series Title: Biological and Medical Physics, Biomedical Engineering.
